# Determining an Evidence Base for Particular Fields of Educational Practice: A Systematic Review of Meta-Analyses on Effective Mathematics and Science Teaching

**DOI:** 10.3389/fpsyg.2022.873995

**Published:** 2022-04-25

**Authors:** Maximilian Knogler, Andreas Hetmanek, Tina Seidel

**Affiliations:** Department of Educational Sciences, TUM School of Social Sciences and Technology, Technical University of Munich, Munich, Germany

**Keywords:** meta-analyses, systematic review, evidence-based/evidence-informed practice, Science Technology Engineering Mathematics (STEM), teaching effectiveness

## Abstract

The call for evidence-based practice in education emphasizes the need for research to provide evidence for particular fields of educational practice. With this systematic literature review we summarize and analyze aggregated effectiveness information from 41 meta-analyses published between 2004 and 2019 to inform evidence-based practice in a particular field. In line with target specifications in education that are provided for a certain school subject *and* educational level, we developed and adopted a selection heuristic for filtering aggregated effect sizes specific to both science and mathematics education *and* the secondary student population. The results include 78 context-specific aggregated effect sizes based on data from over one million students. The findings encompass a multitude of different teaching strategies, most of which offer a measurable advantage to alternatives. Findings demonstrate that context-specific effect size information may often differ from more general effect size information on teaching effectiveness and adherence to quality standards varies in sampled meta-analyses. Thus, although meta-analytic research has strongly developed over the last few years, providing context-specific and high-quality evidence still needs to be a focus in the field of secondary mathematics and science teaching and beyond.

## Introduction

Educational science is a comparably young and dynamic research field. Despite ongoing discussions on the merits and demerits of research in this field, it is remarkable how research activities and applied methodologies have developed over the last few decades (Hedges, 2018). For example, recent years have witnessed a surge of empirical studies on teaching and its associations with learning (Seidel and Shavelson, 2007; Schneider and Preckel, 2017). Simultaneously, there is a greater demand from policymakers that educational policy and practice must be guided by evidence of effectiveness (e.g., No Child Left Behind Act, 2002; Every Student Succeeds Act, 2015).

Due to these developments, it is increasingly imperative for educators as well as policymakers to obtain reliable and accessible information of “what works” in education. Yet, given the proliferation of educational research output and potential evidence that stems from diverse disciplines and methodologies, this is a challenging task. In order to address this challenge and to render the best available evidence usable as a resource, the question of how these research findings can be selected and organized in a specific evidence base is paramount.

Through this systematic review, we address the need for research to provide evidence for evidence-based practice with regard to particular fields of educational practice. The determination of such an evidence base is a multiple step process. In a first step we identify secondary mathematics and science teaching as a particular field of educational practice. Here, we highlight the fact, that goals in teaching at schools are provided on the level of a certain subject and educational level (e.g., Common Core State Standards; Next Generation Science Standards), and conclude that effectiveness information that cuts across these two categories for specification is best suitable for informing effective teaching. In a second step, we then develop a heuristic for selecting the best available evidence for informing decisions within this particular field of practice. In a third step, we operationalize and apply the selection heuristic and analyze the findings by describing the state of accumulated knowledge relevant for this field. Finally, we provide some reflections and suggestions for the further development of this evidence base.

### Evidence for Particular Fields of Educational Practice: Secondary Mathematics and Science Teaching

There has been a growing consensus in numerous countries regarding the general importance as well as specific goals of science and mathematics education (OECD, 2019), which has resulted in the development of (national) educational standards (e.g., Common Core State Standards; Next Generation Science Standards). These standards identify concepts, ideas, and practices that must be emphasized in schools and provide clear normative criteria for successful education in these subjects. It must be noted that educational standards do not merely provide orientation but they are also a core instrument in standards-based reforms aimed at improving educational outcomes. It is on the basis of certain standards that student achievement is assessed and that educators are held accountable for ensuring that their students meet the standard requirements. Importantly, however, standards do not specify effective means for teachers to attain these goals with their students. Thus, identifying effective teaching strategies is one of the hallmark tasks of empirical educational research (Shavelson and Towne, 2002; Mayer, 2004; Hattie, 2009).

Over the last decade, research in science and mathematics education has been particularly productive in terms of collecting high-quality empirical information regarding effective teaching in these subjects (Cheung et al., 2017; Hedges, 2018; Lin et al., 2019). However, the rapid development in scholarship in STEM education has produced an enormous number of studies published in a wide range of journals (Li et al., 2020). The underlying research of these studies is complex as it covers different subjects, grade-levels, student outcomes, among others, and relies on a multitude of different qualitative and quantitative methodological approaches (Brown, 2012; Li et al., 2020). Consequently, this body of empirical research remains rather fragmented, and for educators, it remains unclear what kind of research and which research outlets to consult in order to find out which teaching strategies^1^ they can employ to ensure that students will succeed in meeting the set standards (Kloser, 2014; Cheung et al., 2017). In other words, there is a clear mismatch between the specific, agreed-upon, and easy-to-access information available on binding standards and targets of teaching and an increasing number of scientific literature, which includes complex information on the effectiveness of practices related to reaching those targets. Moreover, compared to the consensus in goals, there seems to be much more diversity regarding a consensus in effective strategies. This lack of consensus is considered one of the main obstacles in addressing calls for evidence-based teaching and in the further advancement of teacher preparation and professional development (Grossman et al., 2009; Windschitl et al., 2012; Kloser, 2014; Lynch et al., 2019), as well as in improving the outcomes of education in general (Cohen et al., 2018). Consequently, the current situation in mathematics and science education both enables and requires working on an evidence base for this particular field of practice.

### Selecting Evidence for a Particular Field of Educational Practice

Determining an evidence base for a particular field of practice is a process of information selection based on well-considered specifications. Some of these specifications are substantive; they define the field of practice for which research findings can serve as warrants in evaluation, decision-making, reflection, and so on (see Cain et al., 2019). Other specifications are methodological; they define the research method that generated the finding (and thus determines its weight as a warrant). In the ensuing paragraphs, we further elaborate on substantive and methodological specifications with regard to the aim of this systematic review—that is, to identify an evidence base for secondary mathematics and science education.

Substantive specifications follow the logic of effective practice (in teaching) including its goals—for example, in terms of educational standards. In simple terms, this logic can be stated in the following manner: an effective teaching strategy X leads to changes in a learning outcome Y in population Z. As highlighted above, educational standards are specific regarding Y and Z (and non-specific regarding X). Standards define learning outcomes in certain subjects and for certain levels of schooling, which in our case are outcomes related to mathematics and science education on the secondary schooling level. Empirical studies in educational research specify all three parameters (and many more). Thus, for establishing an evidence base on effective mathematics and science teaching for the secondary population, it is important to identify research on effective strategies that includes outcomes related to mathematics and science education on the secondary level. This is already a strong limiting factor compared to the vast sources of potentially relevant information. Nevertheless, the resulting evidence base still includes a diverse set of learning outcomes (knowledge, specific and generic skills, attitudes, etc.). Moreover, the evidence base also includes a diverse set of teaching strategies (e.g., inquiry-based teaching), which are often linked to specific outcomes and have previously been categorized on the level of practices (e.g., Bisra et al., 2018), interventions (e.g., Donker et al., 2014), and programs (e.g., Cheung and Slavin, 2013). Thus, although these substantive specifications considerably narrow the scope of eligible research, there is still a lot of diversity in selected evidence, which further calls for a systematic organization of findings in order to support their inclusion for an evidence base.

Methodological specifications result from the properties of the underlying research paradigm (i.e., educational/teaching effectiveness research) and the methodological prerequisites underlying claims for effectiveness. Thus, while the substantive specifications generally define the parameters (X, Y, and Z), methodological specifications pertain to the relationships among these parameters. Again, simply stated, the applied research methodology must support both claims for causality (X causes Y) and claims for (causal) generalizability (X causes Y and this is true for Z). There is considerable consensus that claims for causality are best supported by experimental research (e.g., Shadish et al., 2002), which is characterized by high internal or statistical-conclusion validity. Internal validity depends on a number of factors (type of experimental design, assignment procedure, fidelity of implementation, elimination of experimental confounds, etc.), which often are not optimally realized in teaching effectiveness research (Slavin, 2008, 2020). However, a more general weakness of the experimental approach is the generalizability of this causal relationship (causal generalizability), as most experiments rely on non-representative samples (e.g., convenience samples) of populations and replications are rare. Both aspects reduce the external validity of a study, and the extrapolation of findings from a study to an inference population is often not warranted. Therefore, in the general field of psychology, researchers have proposed measures to increase causal validity in research (e.g., Staines, 2008), and these have been echoed in educational research (e.g., Robinson et al., 2013). With regard to primary studies, authors have encouraged researchers to better address factors that increase internal validity (Shavelson and Towne, 2002; Robinson et al., 2013), which led to a broader implementation of more rigorous research designs in education (Hedges, 2018). Moreover, causal generalizability increases when an effect is found to be present in more than one study (conceptual replication). The effects of the same or of a similar intervention from multiple studies lead to the aggregation of effect sizes. Aggregated effect size estimates are superior to individual studies with respect to replication probability (e.g., Hedges, 2013), and they enable correction of the distorting effects of different error types (e.g., sampling error, measurement error) that often produce the illusion of conflicting findings. Thus, from a methodological perspective, effectiveness claims for the field of teaching are currently best supported by aggregated findings from experimental research. With regard to the process of research to practice transfer, Schraw and Patall (2013, p. 364) also more generally argue that “good practice does not always follow directly from good research, but usually is mediated by synthesis of findings.” Hence, in order to identify the best available evidence for this particular field of practice, we propose considering both substantive and methodological specifications by pooling aggregated effect sizes from experimental research on teaching effectiveness that are specific regarding outcomes and the inference population.

### The Present Review

With this systematic review of meta-analyses, we aim to make a valuable contribution toward creating an evidence-base in a particular field of educational practice. While recent systematic reviews of meta-analytic research provide broad and inclusive summaries (e.g., Hattie, 2009; Schneider and Preckel, 2017), this review seeks to harness the power of focus with regard to the scope and content of analysis. In order to match the level of specificity of educational goals and standards that are both domain *and* schooling-level specific, we seek to develop an evidence base on effective teaching strategies in mathematics and science subjects for secondary student populations. This also takes into consideration that context variables (such as domain and schooling-level) can have considerable impact on the effectiveness of particular teaching strategies (e.g., ; Seidel and Shavelson, 2007; Dignath and Buttner, 2008; Dunlosky et al., 2013; Donker et al., 2014). Due to its strict focus and selection criteria, our approach is limited in that it cannot utilize the full range of knowledge provided by a broader selection of meta-analyses in the field of teaching effectiveness and by the single studies cited therein. Moreover, although we highlight this selective information as particularly relevant for an evidence base, we do acknowledge that there are also other forms of evidence that can or must inform decision-making such as multiple types of data (e.g., Howe, 2009; Windschitl et al., 2012; Dunlosky et al., 2013; Kloser, 2014). Overall, this review closes a gap by providing and analyzing effectiveness information for evidence-based practice specifically in a particular field of educational practice.

For systematic selection and analysis, we developed a selection heuristic which enabled us to filter all meta-analyses that provide at least one aggregated effect size specific to mathematics and science domains and the secondary student population. Our research interest was fourfold. First, we were interested in the number of aggregated effect sizes that are specific to the context of secondary mathematics and science teaching and the particular foci and design of published meta-analyses that provide this information. To this end, we extracted all aggregated effect size estimates matching our selection criteria and described the design of the meta-analyses. Second, we wanted to know to what extent context-specific effect size estimates (for the secondary mathematics and science population) differ from more general effect size estimates (overall effects) reported in selected meta-analyses on teaching effectiveness. If overall effects do not differ from context-specific effects, this may provide some indication that overall effects can provide some orientation for judging the effectiveness of teaching strategies, particularly when more specific effect estimates are not available. Third, in a bottom-up approach, we identified major types of teaching strategies and categorized all aggregated effect sizes from our selected sample into coherent categories (such as inquiry learning or self-regulated learning). This categorization offers a clear and integrated summary of effectiveness information that is both reliable and relevant for the context of secondary mathematics and science education. It enables educators and researchers in the field of effective mathematics and science teaching to estimate the stage of accumulated knowledge, which they can use to further advance work in this field. Fourth, we wanted to analyze the extent to which meta-analyses in the field of mathematics and science teaching currently meet standards for high-quality meta-analytic research. Thereto, we identified established quality criteria from the literature and rated meta-analyses in our sample against these criteria. Findings regarding quality can help to further raise the standard for meta-analyses in educational effectiveness research and thus contribute to a more transparent and reliable evidence base.

## Methods

### Search and Selection

Until May 2019, we systematically searched databases and relevant individual educational review and science and mathematics education journals. We utilized a search string that combined the term “meta-analysis” with further specifications such as “learning,” “teaching,” “teaching effectiveness,” “STEM subjects,” “mathematics,” “science,” “biology,” “physics,” “chemistry,” “secondary education population,” and “student learning outcomes.” We used several approaches to locate relevant literature, including database search (Web of Science, Scopus, ERIC, PsycINFO, and Psych Index), hand-search in top review and (science and mathematics) educational journals, and adopted an ancestral approach by scanning the reference lists of identified publications for further relevant publications. We supplemented all details on the databases, search strings, and the complete list of hand-searched journals (see Supplementary Material S1). The selection process covered two steps: first, the first two authors scanned titles and abstracts for relevance (agreement: Cohen's kappa = 0.65; disagreements were resolved by discussion). Second, from the remaining publications, we assessed full texts in detail for a match to the following eligibility criteria:

1. The study is a meta-analysis, that is, averaged at least two standardized effect sizes obtained from different samples.
2. The meta-analysis analyzed studies^2^ on teaching effectiveness, which include interventions that manipulated an independent variable.
3. The meta-analysis included a student-level outcome measure as a dependent variable.
4. The meta-analysis reported at least one separate effect size specific for secondary education AND mathematics and science subjects.^3^
5. The search filter of the meta-analysis was not explicitly limited to a specific subgroup of students (e.g., students with special needs, low socioeconomic status, gifted students, at-risk students).
6. The meta-analysis was published in a peer-reviewed journal.
7. The meta-analysis was published in or after the year 2004 (cut-off year of inclusion by previous research synthesis: Seidel and Shavelson (2007) and Hattie (2009).
8. The report must be available in English.

**Figure 1:**
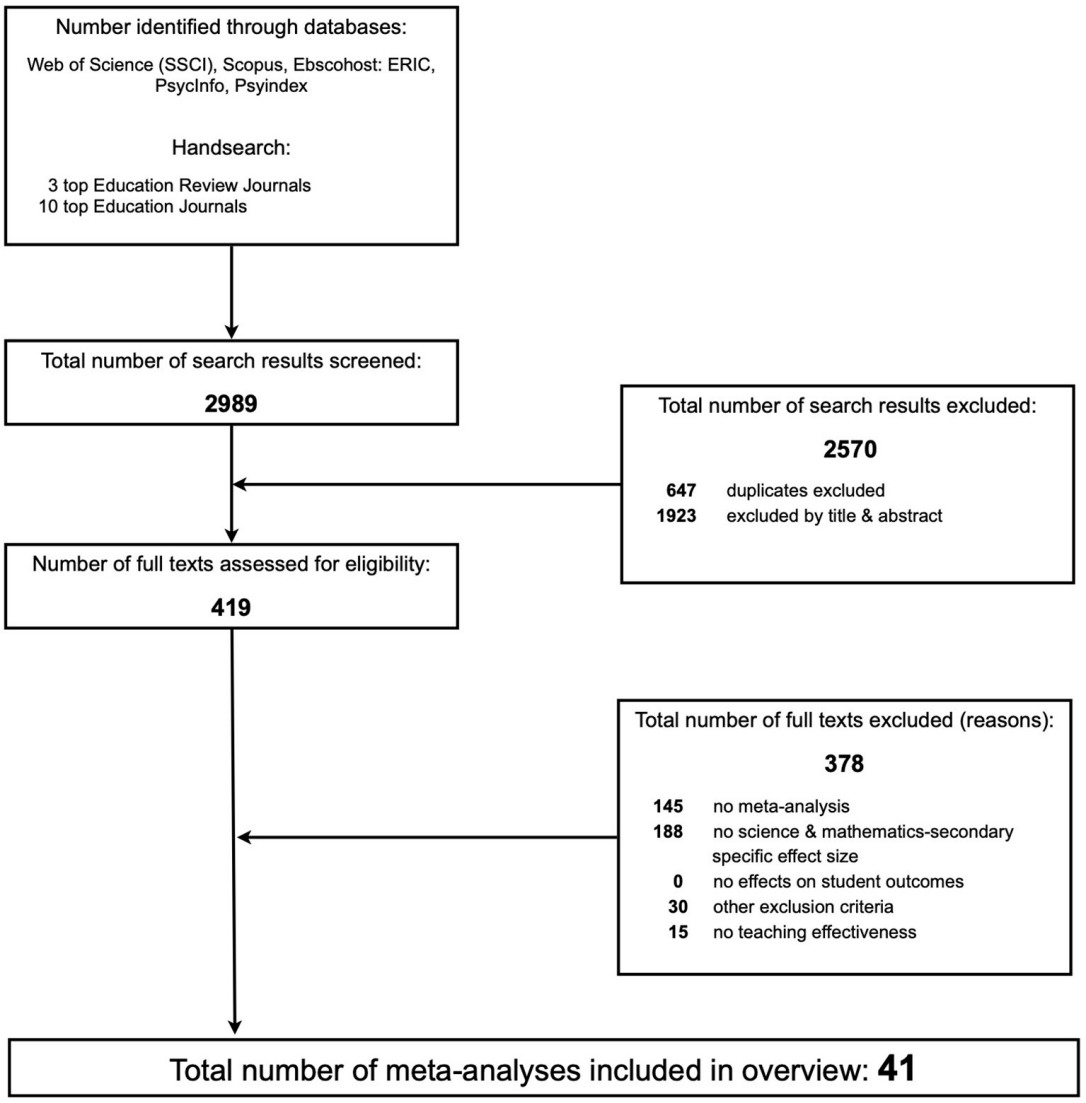
PRISMA flow diagram.

We double coded each study: Cohen's kappa = 0.63 to 1.00 (Mean = 0.77) and inconsistencies were resolved by discussion. In case of missing or insufficient information, we contacted the first authors. Figure 1 depicts the details of the selection process.

### Data Extraction, Coding, and Analysis

#### Procedures

For data extraction and coding, we created an extensive coding manual. All sections of the manual build on existing literature (details in the descriptions below) and underwent a cyclical process of testing, coder training, reliability checks, and adaptation. Using the final version of the manual, the two first authors coded all sampled meta-analyses. Further, agreement rates were checked for each item, and inconsistencies were resolved by discussion. The complete coding manual is pre-registered and together with Supplemental Material provided on Open Science Framework (Weblink: https://osf.io/9n99n/?view_only=bb30c83e9bf34d73a79138ddcf91da5c).

#### Extraction of Effect Sizes

Generally, we extracted effect sizes based on random-effects models (Hedges and Vevea, 1998), including 95% confidence intervals (CI) and the underlying number of primary effect sizes (k). In line with the goal of this systematic review, we extracted all effect sizes specific to both subject-domain (i.e., mathematics, science) and schooling level (i.e., secondary students from middle and high school), as well as overall effects reported in the selected meta-analyses. We consider these specific effect sizes to provide the best available estimate for the context-specific effectiveness of a particular teaching strategy. In order to extract these specific effect sizes, we followed the heuristic depicted in Table 1. Meta-analyses that fulfill our eligibility criteria fall into four categories, depending on their focus of investigation. Meta-analyses belonging to the first category investigate mathematics and science interventions within the secondary student population. These meta-analyses only include primary studies conducted with secondary students in mathematics and science education. All effect sizes included in these meta-analyses are automatically specific and, thus, were extracted. Meta-analyses in the remaining categories are more inclusive (i.e., different educational levels and/or subject domains) and thus use standard methods such as subgroup-analysis or meta-regression (Borenstein et al., 2011) to test for generalizability to the context of secondary mathematics and science education. Thus, the extraction of effect sizes in categories 2–4 meta-analyses can be limited due to restrictions because of a statistically significant moderator influence. For example, if a meta-analysis in category 2 yielded a statistically significant moderating effect of level of schooling, we only extracted the effect size(s) relevant for the secondary level, as only this/these effect size(s) is/are specific for both mathematics and science as well as secondary students. However, if a meta-analysis in category 2 yielded a statistically non-significant moderation by schooling level, we inferred that all effects are robust with regard to the level of schooling. Consequently, we extracted all effect sizes reported in this meta-analysis. The first two authors double coded each meta-analysis that met the above criteria. The rate of agreement was 92%, and the remaining differences were discussed and resolved.

**Table 1:** Heuristic for extracting effect sizes specifically for secondary mathematics and science teaching.

**Category**	**Focus of meta-analysis**	**Moderating effects results**	**Extraction of effect sizes**	**Code**
1	Mathematics and science interventions within secondary student population		All effect sizes extracted	1
2	Mathematics and science interventions with schooling level as moderator	Schooling level sign	Secondary level effect size extracted	2
		Schooling level n.s.	All effect sizes extracted	3
3	Secondary school interventions with subject domain as moderator	Subject domain sign.	Mathematics and science effect size(s) extracted	4
		Subject domain n.s.	All effect sizes extracted	5
4	Teaching interventions with subject domain and schooling level as moderators	Subject domain n.s. + schooling level n.s.	All effect sizes extracted	6
		Subject domain sign. + schooling level sign.	No effect size extracted (publication excluded)	7
		Subject domain sign. + schooling level n.s.	Mathematics and science effect size(s) extracted	8
		Subject domain n.s. + schooling level sign.	Secondary effect size extracted	9

*sign., significant; n.s., not significant; Code: listed for matching information with Table 2*.

#### Comparison of Overall and Specific Effect Sizes

Since the meta-analyses in our sample include both a specific aggregated effect size (often based on a subset of the primary data) as well as overall effects (based on all primary data), we analyzed the extent to which overall effects differ from specific effects in order to determine whether overall effects in general provide good orientation in cases in which more specific effect estimates are not available. To compare specific and overall effects, we extracted all reported overall effect sizes and analyzed the difference between the overall and the specific effect sizes. We thereby distinguished between four levels of difference (see, e.g., Fan et al., 2017): (0) no difference: numeric values of the two-point estimates of the statistical means are identical; (1) weak level of difference: numeric values of the two-point estimates of the statistical means are not identical; (2) moderate level of difference: at least one-point estimates of the statistical mean is not encompassed by the 95% confidence interval of the other mean; (3) high level of difference: 95% confidence intervals of the two-point estimates of the means do not overlap.

#### Analysis of Context-Specific Effectiveness

As a next step in the analysis, the selected effectiveness information was categorized and summarized in a meaningful manner. A particular challenge was given by the heterogeneity of the study characteristics. Although almost all sampled meta-analyses are exclusively based on experimental research to determine the effectiveness of educational interventions on student achievement, our sample demonstrates considerable variations on many parameters that have shown to influence effect sizes (e.g., Slavin and Madden, 2011; de Boer et al., 2014; Cheung and Slavin, 2016). This simultaneous variation on several parameters, particularly in research methodology (e.g., sampling, group assignment, comparison condition, outcome measure, effect size calculation, etc.), complicates comparing and contrasting results across different meta-analyses. The resulting complexity of effect size comparisons, highlighted in the literature (see e.g., Coe, 2002; Hill et al., 2008; Ferguson, 2009; Dunlosky et al., 2013; Belland et al., 2017; Schneider and Preckel, 2017; Simpson, 2018), does not favor rank-ordering effect sizes on a single scale in terms of their magnitude. Thus, instead of providing rank orders, we categorized all aggregated effect sizes into coherent categories with regard to meta-analytic design, teaching strategies, and learning outcomes (see Table 2).

**Table 2:** Effectiveness summary.

**References**	**Code**	**Quality**	**Type of effect size**	**Independent variable (overall effect)**	**Dependent variable (overall effect)**	**k**	**ES**	**CI -/+**	**Independent variable (specific effect)**	**Dependent variable (specific effect)**	**k**	**ES**	**CI -/+**	**ES diff**
**Effectiveness of individual strategies**														
**Inquiry-based and project-based learning**														
Furtak et al. (2012)	1	45%	Glass' d	Inquiry-based science teaching	Science achievement	37	0.50	0.27; 0.73	Inquiry-based science teaching	Science achievement	37	0.50	0.27; 0.73	n.a.
Lazonder and Harmsen (2016)	3	74%	Cohen's d	Guidance in inquiry-based learning	Learning activities	20	0.66	0.44; 0.88	Guidance in inquiry-based learning	Learning activities	20	0.66	0.44; 0.88	n.a.
	3		Cohen's d	Guidance in inquiry-based learning	Performance success	17	0.71	0.52; 0.90	Guidance in inquiry-based learning	Performance success	17	0.71	0.52; 0.90	n.a.
	3		Cohen's d	Guidance in inquiry-based learning	Learning outcomes	60	0.50	0.37; 0.62	Guidance in inquiry-based learning	Learning outcomes	60	0.50	0.37; 0.62	n.a.
Chen and Yang (2019)	8	71%	Hedges‘ g	Project-based learning	Academic achievement	30	0.71	0.67; 0.75	Project-based learning	Academic achievement	11	0.64	0.54; 0.75	1
**Game-based learning**														
Wouters et al. (2013)	8	69%	Cohen's d	Game-based learning	Learning	77	0.29	0.17; 0.42	Game-based learning	Learning in biology	28	0.11	−0.11; 0.33	1
									Game-based learning	Learning in math	16	0.17	0.07; 0.28	1
	6		Cohen's d	Game-based learning	Motivation	31	0.26	−0.03; 0.56		Motivation	31	0.26	−0.03; 0.56	n.a.
	6		Cohen's d	Game-based learning	Retention	17	0.36	Not reported		Retention	17	0.36	Not reported	n.a.
Wouters et al. (2013)	8	69%	Cohen's d	Instructional support in GBL	Learning outcomes	107	0.34	not reported	Instructional support in GBL	Learning outcomes in biology	35	0.59	0.38; 1.76	1
									Instructional support in GBL	Learning outcomes in math	11	0.40	0.10; 1.19	1
Tokac et al. (2019)	3	69%	Hedges'd	Game-based learning	Mathematics achievement	39	0.13	0.02; 0.24	Game-based learning	Mathematics achievement	39	0.13	0.02; 0.24	n.a.
**Self-regulated learning/learning strategies training**														
Dignath and Buttner (2008)	4	56%	Weighted es	SRL training characteristics	Performance	357	0.69	not reported	SRL training characteristics	Performance math secondary	12	0.23	0.07; 0.38	1
de Boer et al. (2014)	8	73%	Hedges' g	Attributes of interventions	Academic performance (math and science)	95	0.66	0.56; 0.76	Attributes of interventions	Academic performance (math and science)	95	0.66	0.56; 0.76	n.a.
Donker et al. (2014)	8	69%	Hedges' g	SRL instruction	Academic performance (math and science)	180	0.66	0.56; 0.76	SRL instruction	Academic performance math	44	0.66	Not reported	0
										Academic performance science	9	0.73	Not reported	1
Bisra et al. (2018)	6	56%	Hedges' g	Self-explanation prompts	Cognitive learning outcomes	69	0.55	0.45; 0.65	Self-explanation prompts	Cognitive learning outcomes	69	0.55	0.45; 0.65	n.a.
Lee et al. (2018)	3	45%	Cohen's d	Metacognitive training	Algebraic reasoning	21	0.97	0.88; 1.06	Metacognitive training	Algebraic reasoning	21	0.97	0.88; 1.06	n.a.
Zheng (2016)	6	60%	Cohen's d	SRL scaffolds in computer-based learning environments	Academic performance	29	0.44	0.23; 0.65	SRL scaffolds in computer-based learning environments	Academic performance	29	0.44	0.23; 0.65	n.a.
**Educational technology: software/individualized learning**														
Li and Ma (2010)	2	74%	Cohen's d	Computer technology	Math achievement	85	0.28	0.13; 0.43	Computer technology	Math achievement	37	0.61	0.43; 0.79	2
Cheung and Slavin (2013)	3	74%	Weighted ES	Technology applications	Math achievement	74	0.16	0.11; 0.20	Technology applications	Math achievement	74	0.16	0.11; 0.20	n.a.
Ma et al. (2014)	6	62%	Hedges' g	Intelligent tutoring systems	Learning outcomes	107	0.41	0.34; 0.48	Intelligent tutoring systems	Learning outcomes	107	0.41	0.34; 0.48	n.a.
Steenbergen-Hu and Cooper (2013)	3	74%	Hedges' g	Intelligent tutoring systems	Math learning	17	0.01	−0.10; 0.12	Intelligent tutoring systems	Math learning	17	0.01	−0.10; 0.12	n.a.
Gerard et al. (2015)	6	57%	Hedges' g	Automated adaptive guidance	Academic achievement	24	0.34	0.23; 0.45	Automated adaptive guidance	Academic achievement	24	0.34	0.23; 0.45	n.a.
	6		Hedges' g	Advanced vs. Simple adaptive guidance	Academic achievement	29	0.27	0.15; 0.38	Advanced vs. Simple adaptive guidance	Academic achievement	29	0.27	0.15; 0.38	n.a.
Belland et al. (2017)	2	86%	Hedges' g	Computer-based scaffolding	Cognitive outcomes	333	0.46	0.37; 0.55	Computer-based scaffolding	Cognitive outcomes: middle school	108	0.37	0.28; 0.48	2
									Computer-based scaffolding	Cognitive outcomes: secondary school	53	0.48	0.35; 0.60	1
**Educational technology: hardware/mobile learning**														
Sung et al. (2016)	9	62%	Hedges' g	Integrating mobile devices with teaching	Academic achievement	108	0.52	0.43; 0.61	Integrating mobile devices with teaching	Academic achievement: secondary school	20	0.45	0.24; 0.66	1
Tingir et al. (2017)	8	76%	Cohen's d	Mobile devices	Achievement	23	0.48	0.26; 0.71	Mobile devices	Math achievement	3	0.16	−0.55; 0.87	2
										Science achievement	8	0.53	0.40; 0.66	1
Sung et al. (2017)	6	57%	Hedges' g	Mobile computer-supported-collaborative learning	Learning outcomes (achievement, attitude, peer-interaction)	163	0.52	0.38; 0.66	Mobile computer-supported-collaborative learning	Learning outcomes (achievement, attitude, peer-interaction)	163	0.52	0.38; 0.66	n.a.
**Design of learning material**														
Ginns et al. (2013)	6	63%	Cohen's d	Conversational style instructional text	Retention	30	0.30	0.18; 0.41	Conversational style instructional text	retention	30	0.30	0.18; 0.41	n.a.
					Transfer	25	0.54	0.25; 0.83		Transfer	25	0.54	0.25; 0.83	n.a.
Schneider et al. (2018)	8	89%	Hedges' g	Signaled multimedia material	Retention	139	0.53	0.42; 0.64	Signaled multimedia material	Retention in biology	32	0.35	0.11; 0.59	2
										Retention in chemistry	4	0.80	0.15; 1.45	2
										Retention in math	9	0.08	−0.32; 0.49	2
										Retention in physics	36	0.43	0.21; 0.65	1
										Retention in geography	17	0.61	0.31; 0.92	1
	6			Signaled multimedia material	Transfer	70	0.33	0.22; 0.43	Signaled multimedia material	Transfer	70	0.33	0.22; 0.43	n.a.
Schroeder and Cenkci (2018)	9	75%	Hedges' g	integrated multimedia design	learning	58	0.63	not reported	Integrated multimedia design	Learning grade 6–8	7	0.43	0.22; 0.63	1
										Learning grade 9–12	7	0.81	0.55; 1.08	1
**Using similarities and differences**														
Apthorp et al. (2012)	6	60%	Hedges' g	Similarities and differences	Achievement (math and science)	14	0.65	0.39; 0.91	Similarities and differences	Achievement (math and science)	14	0.65	0.39; 0.91	n.a.
**Mathematical modeling**														
Sokolowski (2015)	2	71%	Hedges' g	Mathematical modeling	Math achievement	14	0.69	0.59; 0.79	Mathematical modeling	Math achievement high school	7	0.94	0.79; 1.08	3
**Self-grading**														
Sanchez et al. (2017)	6	86%	Hedges' g	Self-grading	Test performance	22	0.34	0.15; 0.52	Self-grading	Test performance	22	0.34	0.15; 0.52	n.a.
**Peer instruction**														
Balta et al. (2017)	8	72%	Cohen's d	Peer instruction	Learning gains	35	0.94	0.70; 1.17	Peer instruction	Learning gains in physics	15	1.30	0.88; 1.71	2
										Learning gains in math	6	0.91	0.41; 1.4	1
										Learning gains in biology	4	0.78	0.48; 1.06	1
										Learning gains in geography	1	0.19	−0.24; 0.63	3
										Learning gains in chemistry	1	0.34	−0.07; 0.75	2
**Homework**														
Fan et al. (2017)	2	91%	Weighted r	Homework	Performance math and science	61	0.22	0.19; 0.25	homework	Performance math and science junior high school	23	0.15	0.11; 0.18	2
										Performance math and science senior high school	17	0.3	0.25; 0.34	1
**Concept maps**														
Schroeder et al. (2017)	6	67%	Hedges' g	Concept maps	Learning	142	0.58	Not reported	Concept maps	Learning	142	0.58	Not reported	n.a.
	6			Concept maps constructed	Learning	75	0.72	0.56; 0.88	Concept maps constructed	Learning	75	0.72	0.56; 0.88	n.a.
	9			Concept maps studied	Learning	67	0.43	0.29; 0.57	Concept maps studied	Learning intermediate level	7	0.82	0.62; 1.02	3
										Learning secondary level	4	1.24	0.79; 1.69	3
**Social and Emotional Learning Programs**														
Corcoran et al. (2017)	3	81%	Hedges' g	School-based social and emotional learning programs	Academic achievement in math	33	0.26	0.18; 0.34	School-based social and emotional learning programs	Academic achievement in math	33	0.26	0.18; 0.34	n.a.
				School-based social and emotional learning programs	Academic achievement in science	5	0.19	0.05; 0.33	School-based social and emotional learning programs	Academic achievement in science	5	0.19	0.05; 0.33	n.a.
**Learning from failure**														
Darabi et al. (2018)	6	64%	Hedges' g	Learning from failure	Learning performance	23	0.43	0.19; 0.68	Learning from failure	Learning performance	23	0.43	0.19; 0.68	n.a.
**Flipped classroom**														
van Alten et al. (2019)	6	92%	Hedges' g	Flipped classroom teaching	Achievement	115	0.36	0.28; 0.44	Flipped classroom teaching	Achievement	114	0.36	0.28; 0.44	n.a.
	6		Hedges' g	Flipped classroom teaching	Satisfaction	22	0.05	−0.23; 0.32	Flipped classroom teaching	Satisfaction	22	0.05	−0.23; 0.32	n.a.
**Comparisons between strategies**														
**Comparisons between innovative approaches**														
Schroeder et al. (2007)	3	62%	Glass' d	Teaching strategies	Science achievement	61	0.67	0.66; 0.68	Teaching strategies	Science achievement	61	0.67	0.66; 0.68	n.a.
Savelsbergh et al. (2016)	2	64%	Pooled d (Morris, 2008)	Innovative teaching strategies	Math & science attitude	60	0.35	0.24; 0.47	Innovative teaching strategies	Math and science attitude	60	0.35	0.24; 0.47	n.a.
	2		Pooled d (Morris, 2008)	Innovative teaching strategies	Math and science achievement	40	0.78	0.60; 0.97	Innovative teaching strategies	Math and science achievement	40	0.78	0.60; 0.97	n.a.
Cheung et al. (2017)	1	52%	Weighted ES	Science programs	Science achievement	21	0.17	Not reported	Science programs	Science achievement	21	0.17	Not reported	n.a.
**Comparisons of instructional methods for learning algebra**														
Haas (2005)	1	26%	Glass' d	Direct instruction	Algebra achievement	19	0.55	0.41; 0.69	Direct instruction	Algebra achievement	19	0.55	0.41; 0.69	n.a.
				Problem-based learning	Algebra achievement	14	0.52	0.35; 0.69	Problem-based learning	Algebra achievement	14	0.52	0.35; 0.69	n.a.
				Manipulatives, models, multiple representations	Algebra achievement	13	0.38	0.28; 0.48	Manipulatives, models, multiple representations	Algebra achievement	13	0.38	0.28; 0.48	n.a.
				Cooperative learning	Algebra achievement	3	0.34	0.30; 0.38	Cooperative learning	Algebra achievement	3	0.34	0.30; 0.38	n.a.
				Communication and study skills	Algebra achievement	5	0.07	0.01; 0.13	Communication and study skills	Algebra achievement	5	0.07	0.01; 0.13	n.a.
				Technology aided instruction	Algebra achievement	12	0.07	−0.10; 0.24	Technology aided instruction	Algebra achievement	12	0.07	−0.10; 0.24	n.a.
Rakes et al. (2010)	1	63%	Weighted ES	New non-technology curricula	Algebra achievement	Not reported	0.40	−0.16; 0.64	New non-technology curricula	Algebra achievement	Not reported	0.40	−0.16; 0.64	n.a.
				Instructional strategies	Algebra achievement	Not reported	0.35	−0.21; 0.49	Instructional strategies	Algebra achievement	Not reported	0.35	−0.21; 0.49	n.a.
				Use of manipulatives	Algebra achievement	Not reported	0.34	0.08; 0.60	Use of manipulatives	Algebra achievement	Not reported	0.34	0.08; 0.60	n.a.
				Technology tools	Algebra achievement	Not reported	0.17	−0.03; 0.31	Technology tools	Algebra achievement	Not reported	0.17	−0.03; 0.31	n.a.
				Technology-based curricula	Algebra achievement	Not reported	0.15	−0.46; 0.76	Technology–based curricula	Algebra achievement	Not reported	0.15	−0.46; 0.76	n.a.
**Comparisons of strategies for fostering critical thinking and scientific reasoning**														
Abrami et al. (2015)	6	66%	Hedges' g	Instructional strategies	Critical thinking skills	341	0.30	0.25; 0.34	Instructional strategies	Critical thinking skills	341	0.30	0.25; 0.34	n.a.
Schwichow et al. (2016)	3	86%	Hedges' g	Teaching control- of-variables-strategy	Control- of-variables-strategy skills	226	0.61	0.53; 0.69	Teaching control-of-variables-strategy	Control-of-variables-strategy skills	226	0.61	0.53; 0.69	n.a.
Engelmann et al. (2016)	3	66%	Hedges' g	Interventions on scientific reasoning	Scientific reasoning	30	0.71	0.55; 0.87	Interventions	Scientific reasoning	30	0.71	0.55; 0.87	n.a.

*Code, code of meta-analysis for extracting effect sizes (see Table 1); k, number of raw effect sizes; ES, effect size; CI±, lower and upper limit of the confidence interval of the effect size estimation; specific effect, effect specific for mathematics and science education in secondary schooling level; n.a., not applicable for cases when overall effect equals specific effect; ES diff, difference from the comparison of overall effects with effect sizes specific for mathematics and science education in secondary schooling level (categories are described in the text)*.

#### Analysis of Scientific Quality

In order to enable reproducibility and alleviate threats to validity, researchers in different fields have developed manuals and standard documents that offer guidelines for meta-analysts (e.g., AMSTAR: Shea et al., 2007; APA's Meta-Analysis Reporting Standards (MARS), PRISMA: Moher et al., 2009). In addition to handbooks (e.g., Borenstein et al., 2011; Cooper, 2015; Higgins et al., 2019) and recent scientific evaluations of meta-analytic practice (e.g., Ahn et al., 2012; Cooper and Koenka, 2012; Polanin et al., 2017; Schalken and Rietbergen, 2017; Siddaway et al., 2019), these provide a strong resource to ensure the scientific quality of meta-analytic work. Moreover, systematic reviews are in danger of accumulating bias and error when the methods utilized at the level of included meta-analyses and primary studies are not evaluated (e.g., Polanin et al., 2017). Since researchers have noted a wide variation in transparent reporting and employing sound methodologies (Ahn et al., 2012), we analyzed all 41 publications in terms of their implementation of strategies to avoid biased findings. Our coding scheme is based on the abovementioned literature review, finally comprising 37 items. It has to be noted that the quality of meta-analyses depends on numerous details and our items do not intend to exhaustively capture all these aspects. However, taken together, these criteria provide a reasonable indication of efforts that have been made to ensure a high quality of scientific information justified by recent literature, even signaling room for improvement. We organized all items in accordance with the guidelines for conducting a meta-analysis of experimental research. These include open science (2 items), search and selection (7 items), coding and data collection (10 items), and meta-analytic methods (18 items). The intercoder agreement for all items ranged from Cohen's kappa = 0.74 to 1.00. Inconsistencies were resolved by discussion. For a detailed description of each item, see Table 3.

**Table 3:** Scientific quality.

**Item**	**Code**	**Item description**	**% of sample**
**Open science**			
Open protocol	q_pr	Is a pre-registered study plan/protocol published? (y/n)	0%
Open data	q_od	Are relevant data for reproducibility of statistical analyses published? (y/n)	44%
**Search and selection**			
Search terms	q_st	Is a complete description of database search terms/full search string provided? (y/n)	93%
Search strategies	q_sp	Were additional search strategies applied? (e.g., hand-search) (y/n)	73%
Exclusion criteria	q_ec	Are inclusion/exclusion criteria clearly stated? (y/n)	100%
Search period	q_spr	Is information about search period provided? (y/n)	95%
NPR publications included	q_pi	Are effect sizes from non-peer-reviewed (NPR) publications included? (y/n)	71%
Selection reliability	q_sr	Is an indicator for selection reliability provided? (y/n)	34%
List of included publications	q_lip	Is a complete list of included publications provided? (y/n)	98%
**Coding and data collection**			
Sample description	q_ip	Is the sample population for each included primary study specified? (y/n)	54%
Intervention description	q_ii	Is the intervention for each included primary study specified? (y/n)	78%
Control description	q_ic	Are control conditions for each included primary study specified? (y/n/na)	55%
Outcome description	q_io	Are outcome variables for each included primary study specified? (y/n)	51%
Outcome statistics	q_rs	Are descriptive statistics for outcome variables reported? (y/n)	41%
Study design	q_id	Is the study design for each included primary study specified? (y/n)	41%
Coding process	q_cp	Is the coding/data collection process described? (y/n)	78%
Coder qualification	q_cq	Is the qualification of coders reported? (y/n)	49%
Coding categories	q_cd	Are coding categories for all variables clearly defined? (y/n)	85%
Coding reliability	q_cr	Is an indicator for coding reliability provided? (y/n)	80%
**Meta-analytic methods**			
Missing data handling	q_hdm	Is a procedure for handling of missing data described? (y/n)	71%
Effect size description	q_esd	Is there a verbal description of how raw effect sizes are determined? (y/n)	95%
Effect size calculation	q_esc	Is an exact formula for the calculation of raw effect sizes reported? (y/n)	39%
OAE: Statistical model	q_rm	Is a statistical model for the overall effect size estimation (OAE) reported? (y/n/na)	92%
OAE: Model justification	q_jm	Is a justification for the statistical model selection of the OAE provided? (y/n/na)	89%
OAE: Confidence intervals	q_rci	Are confidence intervals for OAE reported? (y/n/na)	90%
ME: Statistical model	q_rma	Is a statistical model for moderator effect size estimation (ME) reported? (y/n/na)	97%
ME: Model justification	q_jmm	Is a justification for the statistical model selection for ME provided? (y/n/na)	95%
ME: Confidence intervals	q_rcm	Are confidence intervals for ME reported? (y/n/na)	95%
ME: Multiple moderators	q_rmm	Is the issue of multiple moderator tests discussed? (y/n/na)	48%
BSV: indicator	q_rabv	Is an indicator for the quantity of between-study variance (BSV) reported? (y/n/na)	89%
BSV: estimation	q_rmbv	Is an exact formula for the estimation of between-study variance reported? (y/n/na)	50%
Dependent measures	q_rdm	Is a procedure for handling dependent data points reported? (y/n/na)	79%
Application of HLM	q_aa	Is hierarchical linear modeling applied for dependent data points? (y/n/na)	60%
Statistical power	q_stpr	Is a statistical power analysis reported? (y/n)	5%
Publication bias	m_pb	Is a publication bias test reported? (y/n)	83%
Outlier sensitivity analysis	m_os	Is an outlier sensitivity analysis reported? (y/n)	56%
Scientific quality	m_sq	Is an indicator for scientific quality (e.g., standardized measures; study design; publication status) of primary studies used for moderator analysis? (y/n)	61%

*Code (code information for matching with Supplemental Material); y, yes; n, no; na, not applicable (this item was not applicable for an individual meta-analysis and thus, the individual meta-analysis was not included in the percentage score)*.

## Results

### Availability of Specific Aggregated Evidence

A total of 41 meta-analyses published between January 2004 and May 2019 met all our inclusion criteria. In a stepwise process of selection, 378 publications were excluded because they did not meet one or several inclusion criteria. For example, in the second step, 188 publications were excluded because they did not provide a context-specific effect size. Although these publications might have also been omitted because of not meeting other criteria (e.g., not investigating teaching effectiveness; focusing on a particular group of students), this number is still an indication that a substantial number of meta-analyses might not provide context-specific information. With one exception,^4^ all the selected meta-analyses used aggregated d-family effect sizes based on comparisons between specific teaching strategy interventions and alternatives (mostly certain regular or traditional teaching practices as control condition). All publications provided information on the number of studies that were included. In sum, analyses are based on a total of 2,708 (M = 66.05; SD = 104.59)^5^ primary studies reporting 4,594 (M = 112.05; SD = 1,151.99) effect sizes and involving an estimated number of 1,159,143 (M = 28,271.78; SD = 60,438.86) participants. The sampled meta-analyses were published by 17 different peer-reviewed journals and include an average time span of 21 years (SD = 13.61) of primary research (see Supplementary Material S2 for details).

Overall, we extracted 78 aggregated effect sizes specific for both science and mathematics education and the secondary student population that are not disaggregated for other (moderating) variables (i.e., variations in sample population, treatment, method, study context, etc.). These effect sizes provide the most inclusive estimate of context-specific effectiveness (see Table 2). Of these 78 context-specific aggregated effect sizes, 13 (17%) stem from 4 meta-analyses on mathematics and science interventions within the secondary student population (category 1),^6^ 20 (26%) stem from 14 meta-analyses on mathematics and science interventions with schooling level as a moderator (category 2), 1 (1%) stems from 1 meta-analysis on secondary school interventions with school subject as a moderator (category 3), and 44 (56%) stem from 22 meta-analyses on teaching interventions with subject domain and schooling level as moderators (category 4).

In sum, the majority of meta-analyses providing context-specific aggregated effect size estimates in our sample are meta-analyses on teaching interventions across subjects and schooling levels (56% of extracted context-specific effect sizes) and meta-analyses on mathematics and science interventions across different schooling levels (26% of extracted context-specific effect sizes). With 17% of all extracted context-specific effect sizes, context-specific meta-analyses with a focus on mathematics and science subjects as well as the secondary student population provide a relatively small proportion of context-specific effectiveness information.

### Comparison Between Overall and Specific Effect Sizes

Using 78 domain and schooling level-specific aggregated effect sizes that are not disaggregated for other variables, we compared overall and specific effect sizes in the sampled meta-analyses. In 47 cases, the overall effect reported in the meta-analysis is specific for secondary mathematics and science and, thus, represents the best available context-specific effect size. In 31 cases, the overall effect is not specific for secondary mathematics and science. In these cases, we compared the context-specific effect size based on a subsample of primary studies to the overall effect reported in the meta-analysis. Table 2 provides a summary of overall effects, specific effects, and comparison results for all dependent and independent variables. In 1 out of 31 comparisons (3%), the overall effect and context-specific effect have the same numerical value (level 0). Further, 17 out of 31 comparisons (55%) yielded a weak level of difference with numerical values of means being different (level 1); 9 out of 31 comparisons (29%) yielded a moderate level of difference with at least one mean not being covered in the confidence interval of the other mean (level 2); and 4 out of 31 comparisons (13%) yielded a high level of difference with no overlap between the confidence intervals of the two means (level 3). In summary, the majority of comparisons (60%) yielded no or small differences between overall and specific effects, 29% of comparisons resulted in moderate differences, and a small number of comparisons (13%) indicated large differences.

### Summary of Effectiveness Information

Table 2 provides a comprehensive summary of effectiveness information. Row-wise, the table lists all 41 meta-analyses^7^ that matched our selection criteria organized in specific categories (see the following paragraph). Column-wise, the table details information both on the overall effect reported in the publication, which is based on all primary studies (mid columns) of the meta-analyses, and on the aggregated effect size(s) specific for the context of secondary mathematics and science education (right columns). As regards the categorization applied, our analysis showed that sampled meta-analyses follow two major organizing principles: First, most meta-analyses (*N* = 33) are teaching strategy-focused, that is, they analyze the effectiveness of a specific teaching strategy (e.g., inquiry learning, flipped classroom) with regard to one or several student outcomes related to mathematics and science learning (e.g., mathematics/science achievement, student motivation in mathematics/science) (see e.g., Furtak et al., 2012). Second, some meta-analyses (*N* = 8) are outcome-focused, that is, they compare several different teaching strategies (e.g., direct instruction vs. problem-based learning vs. cooperative learning etc.) with regard to a specific student outcome related to mathematics and science learning (e.g., critical thinking, algebraic reasoning). In addition, some sampled meta-analyses focused on similar teaching strategies (e.g., three meta-analyses investigated inquiry project-based learning strategies) or similar student outcomes (e.g., critical thinking and scientific reasoning) and were thus further grouped together.

**Figure 2:**
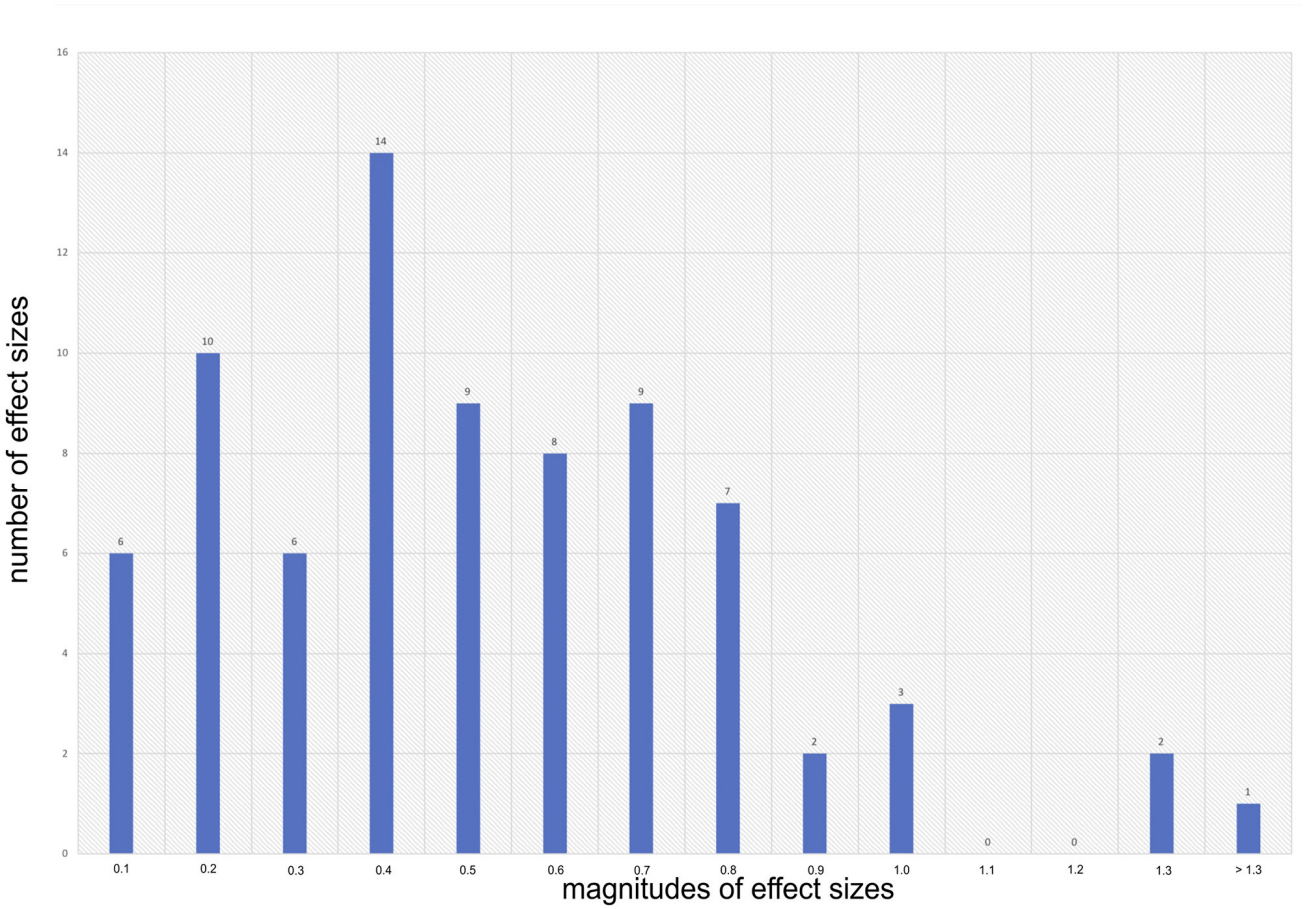
Distribution of effect sizes.

In line with our selection criteria, all sampled meta-analyses provide at least one aggregated effect size estimate specific for the effectiveness of mathematics and science teaching on the secondary level. Without exception, all of these 78 effect sizes are positive. Figure 2 presents the distribution of all context-specific mean effect sizes. Effect size estimates range between ES = 0.01 and ES = 1.3 with 12 effect size estimates transcending conventional thresholds of statistical significance (i.e., 0.95% confidence intervals include the value zero). About 80% of context-specific aggregated mean effect sizes are 0.2 or larger and 54% are 0.4 or larger. Overall, the size of our sample signals that research has accumulated a substantial number of meta-analyses on various teaching strategies and student outcomes related to secondary mathematics and science teaching. With all effect sizes being positive, this research indicates higher aggregated effectiveness of experimental conditions compared to control conditions.

### Scientific Quality of Included Meta-Analyses

In order to provide a concise summary of quality information, we organized scientific quality data in two ways: (a) Table 3 depicts all the quality items that were coded and summarizes the percentage of the 41 meta-analyses that performed and/or reported what was required by this item. (b) The third row of Table 2 reports a summary quality score averaged across all 37 quality items for each meta-analysis individually (see Supplementary Material S3 for details).

On average, sampled meta-analyses fulfilled 68% (*SD* = 13%) of all criteria coded. Table 3 indicates that on 15 items, over 80% of sampled meta-analyses provided sufficient information. With no meta-analysis being pre-registered and less than half (44%) offering sufficient information to reproduce statistical analyses, issues of open science were not adequately addressed. Criteria relating to search and selection mostly achieved high ratings—for example, with all meta-analyses clearly stating inclusion criteria (100%) and 93% providing sufficient information to reproduce the database search. When it comes to transparency of coding and data collection, approximately half of the sampled meta-analyses failed to provide sufficient information on which data they extracted from primary studies [e.g., specification of control condition (55%), outcome variable (51%), and related descriptive statistics (41%)]—for example, by publishing a primary study coding table (Polanin et al., 2020). The category meta-analytic methods yielded mixed results. Numerous issues relating to data aggregation and bias reduction were reported by a majority of meta-analyses. Yet, although 95% verbally describe how they determined raw effect sizes from primary studies, less than half (39%) provide precise formulas, which clearly describe how data from different primary study designs (e.g., comparison of post measures vs. comparison of pre-post gains) were converted into effect sizes. Similarly, most meta-analyses (89%) provide at least one indicator for between-study variance, but only half (50%) report the exact estimation method (Hedges et al., 2010; Borenstein et al., 2011).

Further, although almost all meta-analyses conducted multiple moderator tests, only half of these (48%) discussed issues such as Type 1 error inflation and confounding (moderator) variables (see Cafri et al., 2010). A majority, but not all, of the meta-analyses (83%) tested for publication bias; moreover, although numbers and magnitude of raw effect sizes in moderator analyses are often relatively small, only two meta-analyses (5%) (Corcoran et al., 2017; van Alten et al., 2019) reported retrospective statistical power for the significance test used to determine the number of studies necessary for detecting a statistically significant effect (Hempel et al., 2013). Further, 56% of the meta-analyses scanned their data for outliers, which could have biased the results; 61% of the meta-analyses investigated the moderating effects of at least one scientific quality indicator of the primary studies (e.g., utilization of standardized vs. non-standardized outcome measures), yet none of these included a multidimensional assessment based on a quality assessment tool (see, e.g., Valentine and Cooper, 2008). Table 2 indicates that scientific quality scores of individual meta-analyses ranged from 26% (min) to 92% (max), with half of the meta-analyses having a score lower or higher than 65%. In summary, the majority of sampled meta-analyses adheres to most quality criteria. A high level of scientific quality, however, is not a consistent finding, since some quality criteria are not adequately addressed by many meta-analyses and a few meta-analyses do not meet several important quality criteria.

## Discussion

In order to be successful, educational systems require orientation both in terms of goals as well as in pathways to attain these goals. Numerous countries have been successful in agreeing on common standards and, thus, specifying binding goals for mathematics and science education. As a consequence, pathways to attain these goals must be further specified. Educational research can contribute to attaining these goals by providing information on those pathways that have been revealed to be most effective. If this information is recognized and accounted for by different stakeholders, one of the most important capacities of educational sciences can be used to contribute to an ongoing improvement of educational systems (Kloser, 2014; Slavin, 2020).

This systematic review seeks to provide a systematic analysis and review of aggregated findings within the experimental or quasi-experimental framework for a certain subject domain and a certain educational level. Furthermore, this systematic review investigated to what extent reported effects sizes on an overall level systematically differ from effect sizes particularly determined for the field of secondary mathematics and science teaching. It also outlines to what extent included meta-analyses meet established quality criteria in meta-analysis research. Overall, this contribution complements efforts that seek to identify a set of core or high-leverage practices (Windschitl et al., 2012; Kloser, 2014) in science and mathematics education as well as more general efforts to synthesizing knowledge on effective teaching and learning (e.g., Seidel and Shavelson, 2007; Hattie, 2009; Dunlosky et al., 2013). In the following account, we summarize five major findings and highlight implications to inform future research.

### Research on Secondary Mathematics and Science Teaching Provides a Substantial Amount of Context-Specific Effectiveness Information

Regarding the field of mathematics and science teaching and current meta-analyses in this specific field, our results demonstrate that research offers a substantial number of specific aggregated effect sizes that encompass various kinds of teaching interventions that are relevant for secondary science and mathematics classrooms. We identified 78 aggregated effect sizes from the last 15 years that provide information that is specific to mathematics and science education. A majority of these effect sizes stem from more general meta-analyses on teaching interventions, which include mathematics and science subjects as well as secondary students as subpopulations. However, specific meta-analyses with a focus on mathematics and science teaching (e.g., Furtak et al., 2012) or even an exclusive focus on secondary mathematics and science populations are forthcoming (e.g., Cheung et al., 2017). Summarizing research from the previous decade (until 2004), Seidel and Shavelson (2007) concluded (for this time period) that the underlying primary research on teaching effectiveness was largely dominated by correlational design studies. The included meta-analyses in our current sample demonstrate (for the following 15 years) that experimental research on teaching effectiveness is increasingly available. In a majority of the underlying experimental primary studies, innovative teaching strategies were compared to some form of conventional, traditional, business-as-usual practice. In aggregating these effects, the meta-analyses in our sample generally enable conclusions regarding whether or not and under what circumstances the innovation is more effective than traditional practice. Moreover, this review also demonstrates that in current meta-analytic research, these comparisons are organized in three major ways, which allow for additional conclusions.

First, a minority of included meta-analyses (20%) were focused on a dependent variable specific to mathematics and science education (e.g., critical thinking, scientific reasoning, attitudes toward science), synthesizing all teaching-related research with this variable as a target outcome (“outcome-focused meta-analyses”). This entails that a number of teaching strategies (inquiry learning vs. collaborative learning vs. digital learning etc.) potentially fostering this outcome are included. While Schroeder et al. (2007) focused on achievement as an outcome, several years later, other outcomes (that were less frequently covered in primary research) have been included in meta-analyses. For example, Savelsbergh et al. (2016) collected research on student attitudes toward mathematics and science, which is still a less frequently studied outcome in primary studies. Their meta-analysis (of *k* = 63 studies) includes various teaching strategies such as inquiry learning, digital learning, and collaborative learning. Second, a majority of included meta-analyses (80%) were focused on a specific teaching strategy in the field of mathematics and science teaching as an independent variable (e.g., inquiry learning, game-based learning etc.), synthesizing all outcome-related research (“teaching strategy-focused meta-analyses”). These meta-analyses enable a nuanced analysis of the effectiveness of that strategy under different conditions and for different learning outcomes (e.g., Wouters et al., 2013). Third, researchers are able to shift the focus of not only their meta-analytic investigation from dependent to independent variables but also with regard to the kind of comparison in the underlying primary research. While numerous meta-analyses include primary research that compares some kind of innovative practice to a traditional practice to determine an effect size, a few more recent meta-analyses include primary studies that compare variations of innovative approaches, such as inquiry learning or game-based learning, with vs. without guidance (Wouters et al., 2013; Lazonder and Harmsen, 2016), or simple versions of automated adaptive guidance vs. advanced versions of automated adaptive guidance (Gerard et al., 2015). Thus, following the establishment of the general effectiveness of a certain teaching strategy, research and research synthesis is now moving forward by carefully studying specific features (and their variations), which can render the application of that teaching strategy more effective. Thus, while previous systematic reviews of STEM research mainly documented quantitative growth, for example, in the increasing number of journal publications (see Li et al., 2020), this review shows the cumulative nature of this research.

### Context (Subject Domain/Educational Level) Is Important in Research on Teaching Effectiveness

Since this systematic review seeks to provide context-specific information, we filtered meta-analyses that include aggregated effect sizes for outcomes of secondary mathematics and science teaching. Using this rationale for selection led to the exclusion of numerous meta-analyses that did not offer information that was sufficiently specific for this context. This is not surprising, as specific areas of teaching effectiveness research may not have accumulated a sufficient number of studies for context-specific analysis. Yet another reason lies in the fact that research syntheses are often not undertaken for the sake of providing context-specific effectiveness information in line with a particular field of practice, but rather for synthesizing findings in a particular research area for theory-building and for reaching broad generalizations (Gurevitch et al., 2018).

However, this review highlights a research synthesis perspective for a particular field of practice. Conceptually, it provides a heuristic for formulating specific inclusion/exclusion criteria that are appropriate for selecting context-specific effectiveness information. In this sense, it showcases requirements that can be considered by researchers and meta-analysts in order to generate more context-specific information for evidence-based practice. Empirically, it demonstrates that context is of significance in terms of teaching effectiveness: when comparing context-specific effect sizes with overall effects in our sampled meta-analyses, we observe varying degrees of difference. Although the majority of comparisons (60%) indicated no or small differences, we also observed many instances (40%) with relevant differences (Schauer and Hedges, 2020), in which case, using the overall effect could lead to different conclusions for evidence-based practice as compared to using the context-specific effect size.

In line with previous research (e.g., de Boer et al., 2014), the findings of this review demonstrate that teaching strategies vary in terms of their effectiveness depending on the contextual conditions designated by a certain field of practice (Taylor et al., 2018). This makes a good case for research and research synthesis that generates and provides context-specific effectiveness information. With this review, we hope to create more awareness for these issues so that researchers can take appropriate action, like conducting context-specific meta-analyses that select and synthesize context-specific primary studies (e.g., Hillmayr et al., 2020).

### Standards-Related Targets Are Addressed by Research on Effective Teaching Strategies

Beyond documenting the availability of specific effectiveness information, this review reveals that a variety of outcomes specified by current standards (such as the Framework for K-12 Science education (National Research Council, 2012), the Next Generation Science Standards, or the Common Core Standards in mathematics) can be attained through instruction using a number of effective pathways (de Kock et al., 2004). In the context of the current standards, process skills such as inquiry and argumentation represent broader educational goals that are addressed in literacy conceptualizations. In this context, student attitudes and motivation, both as prerequisites to learning as well as desirable outcomes, are considered as important goals in their own terms (Kuhn, 2007). The research encompassed by this review includes outcomes such as attitudes and interest in science (e.g., Savelsbergh et al., 2016), motivation (e.g., Wouters et al., 2013), inquiry skills (Lazonder and Harmsen, 2016), critical thinking skills (Abrami et al., 2015), control of variables strategy skills (Schwichow et al., 2016), scientific reasoning and argumentation skills (Engelmann et al., 2016), knowledge transfer skills (e.g., Ginns et al., 2013), and skills of knowledge acquisition and self-regulation (Donker et al., 2014). Thus, in addition to traditional outcome measures such as factual knowledge and achievement, a broader range of educational goals, particularly relevant for current mathematics and science curricula, is encompassed in primary studies and synthesized in meta-analyses. Moreover, a few multicriterial investigations in meta-analyses, assessing effectiveness simultaneously for more than one outcome, were able to demonstrate multicriterial effectiveness of a variety of teaching strategies (e.g., Savelsbergh et al., 2016).

Our results also demonstrate that the sampled meta-analyses address goals of varying scope. Certain teaching strategies support specific targets in terms of standards. For example, the teaching strategy “inquiry learning” can support students effectively in acquiring inquiry skills in addition to domain-specific knowledge (Lazonder and Harmsen, 2016). Other strategies are more universal and do not serve so much as general approaches to teaching but as tools to be incorporated into any lesson or instructional unit to foster mathematics and science learning. Teaching strategies such as using concept maps, self-explaining, or self-grading are not merely easy and cost-efficient to integrate, they are also not restricted to a certain specific content but lend themselves to fostering various learning goals related to standards and curricula.

In order to attain complex goals (such as critical thinking skills etc.) set by standards and curricula in secondary mathematics and science education, classroom learning requires the implementation of more open-ended and complex tasks, which place higher demands on students and thus often require adequate guidance. In reviewing the results of this review, guidance seems to be an important element across different teaching strategies. Students involved in problem-based learning, inquiry learning, or game-based learning were able to profit from teacher or software guidance (Furtak et al., 2012; Wouters and van Oostendorp, 2013; Belland et al., 2017). Importantly, effect sizes in comparisons between guided and non-guided versions of these strategies were as high as effect sizes in the basic comparisons between an innovative strategy (i.e., inquiry and game-based) and a traditional approach (Furtak et al., 2012; Wouters et al., 2013; Lazonder and Harmsen, 2016). Thus, in the context of the learner population of secondary students, the increasing complexity of the demands of the curriculum and with practice moving from a teacher-centered to a learner-centered pedagogy, this seems to suggest that guidance is a crucial element for students succeeding on standard targets.

### The Majority of Aggregated Effect Sizes Are Positive

All of^8^ the investigated teaching strategies indicate beneficial effects on student outcomes in terms of positive aggregated mean effect sizes. Although research on the effectiveness of teaching rests on the basic assumption that research-based teaching strategies can be and generally are effective, it may still be surprising that virtually all aggregated effect sizes selected and presented here were positive. In other words, given the wide range of teaching strategies investigated, one might expect some of these strategies on average to have negative effect sizes and not every tested strategy to work well. This review, however, is not the first systematic review on treatment effectiveness in education yielding mostly positive findings. Other research synthesists have found similar results (e.g., Lipsey and Wilson, 1993; Hattie, 2009; Schneider and Preckel, 2017). In their comprehensive review of 320 independent meta-analyses analyzing the efficacy of psychological, educational, and behavioral interventions, Lipsey and Wilson (1993) found almost only positive mean effect sizes. Similarly, Hattie (2009) synthesis of over 800 meta-analyses, which includes 520 individual meta-analyses on the effects of different teaching approaches on student achievement across different educational levels and subject domains, yielded no negative aggregated effect size for any of the included teaching approaches.

More recently, Schneider and Preckel (2017), in their systematic review on variables associated with achievement in the context of higher education, identified only 2 out of 42 aggregated effect sizes (< 5%) indicating a negative association (with all others being positive) between an instructional approach and student achievement with 1 of the 2 effect sizes representing evidence for the seductive detail effect and thus being expected to be negative. Moreover, after testing for different potential biases and finding no indication for a particular upward bias, Lipsey and Wilson (1993) concluded that “the treatment approaches represented in meta-analysis and reviewed in this article represent rather mature instances that are sufficiently well developed and credible to attract practitioners and sufficiently promising (or controversial) to attract a critical mass of research. For treatment approaches meeting these criteria, it is perhaps not surprising that a high proportion do prove at least moderately efficacious” (Lipsey and Wilson, 1993, p.1200). Thus, based on previous systematic reviews of meta-analytic research on educational intervention, our result of all available effect sizes in the context of secondary mathematics and science teaching being positive was to be expected and our results confirm this expectation.

Although, there seems to be no controversy around the positive direction of results of educational or instructional interventions, there is an ongoing controversy about the magnitude of standardized effect sizes as a metric for evaluating and interpreting the effectiveness of educational interventions (de Boer et al., 2014; Cheung and Slavin, 2016; Simpson, 2018). A focal point of this discussion constitutes the numerous factors (including potential biases) that have been shown to influence standardized effect sizes. By adopting a selection heuristic that takes into account effect size variation due to subject domain and educational level, we have filtered for two of these factors (educational level and subject domain) in order to provide a reliable estimate of the effectiveness of educational intervention in the context of secondary mathematics and science teaching (e.g., de Boer et al., 2014). Nevertheless, previous research has documented other and equally important factors that influence results and should be considered when interpreting (aggregated) effect sizes of educational interventions (Cheung and Slavin, 2016; Kraft, 2020).

For example, Cheung and Slavin (2016) examined methodological impacts on effect sizes using a rather homogenous sample of 645 high-quality studies of educational program evaluations across the grades of prekindergarten to 12, involving reading, mathematics, and science. Their results indicate that research design (randomized vs. non-randomized), sample size (small sample size < N = 250 participants < large sample size), outcome measures (researcher-made vs. standardized measures), and type of publication (published vs. non-published) were all independently associated with effect-size magnitude. Consequently, the authors conclude that these factors need to be accounted for by researchers and policy makers before interpreting and comparing effect sizes from program evaluations. Similarly, de Boer et al. (2014), in their meta-analysis of learning strategy interventions, found that four factors related to how interventions were implemented and how effects were examined together explained 64% of the variance in intervention effect size. Clearly, our sampled meta-analyses demonstrate variations on many parameters that have shown to influence effect sizes (e.g., Slavin and Madden, 2011; de Boer et al., 2014; Cheung and Slavin, 2016). This simultaneous variation on several parameters, particularly in but not limited to variations in research methodology (e.g., sampling, group assignment, comparison condition, outcome measure, effect size calculation etc.) both on the level of primary research and on the synthesis-level, complicates interpreting effect sizes as well comparing and contrasting results across different meta-analyses.

Although this systematic review takes into account some of these aspects by filtering aggregations of experimental research in a particular context, it does not provide an in-depth analysis and discussion of all aspects. One reason is that information necessary for such an in-depth evaluation is oftentimes missing or not sufficiently documented in published meta-analyses. We address this issue in our analysis and discussion of scientific quality (see below). Another reason is that each meta-analysis in our sample, despite communalities, represents a specific configuration with regard to study sampling and analysis of teaching effectiveness research. Thus, a thorough analyses and interpretation of findings that does justice to the complexity of such configurations needs to consider each meta-analysis individually, which is beyond the scope of this publication. We agree with many previous researchers (e.g., Coe, 2002; Ferguson, 2009; Schneider and Preckel, 2017; Simpson, 2018; Kraft, 2020) who have cautioned readers not to reach simple conclusions from complex effect-size estimates. To receive some orientation when interpreting effect sizes, readers can consult recent literature (e.g., Kraft, 2020) or team up with trained researchers to reach informed conclusions.

To increase the potential for coherent interpretation and comparison of findings in future research synthesis, meta-analysts need to reduce heterogeneity when sampling primary research. Cheung and Slavin (2013, 2017), for instance, put together a set of inclusion criteria—particularly suited for the study of educational interventions in classrooms—to increase quality and comparability of findings in meta-analytic research in this field (see also Slavin and Lake, 2008). Similarly, Abrami et al. (2015) increased the homogeneity and the quality of sampled primary research by testing the influence of methodological study features on the effect sizes and consequently excluding pre-experimental designs and non-standardized measures from further analyses. Although these strategies depend on the availability of appropriate numbers and sufficient quality or similarity of primary research, they might also encourage and orient researchers to design primary studies in accordance with such criteria and thus contribute to a more homogenous database. Moreover, homogeneity of sampled primary research is also an important prerequisite for decisions regarding implementation of teaching strategies and educational interventions and related discussions about possible benchmarks for implementation. In this context, researchers have advocated empirical benchmarks “for specific classes of studies and outcome types based on the distribution of effect sizes from relevant literature” (Kraft, 2020, p. 247). Consequently, results from research synthesis in education can only appropriately inform the interpretation of intervention effectiveness and implementation decisions, as far as the interpreter considers the fact that aggregated mean effect sizes represent highly compounded information. Effect sizes generated by meta-analytic aggregation are atop a hierarchy that ultimately rests on the individual research design elements of all single primary studies included in the synthesis. Given the large heterogeneity of research included in this review, which is typical for the field, our results defy broad effectiveness conclusions and instead put the spotlight on each individual meta-analysis and aggregated mean effect size(s) reported therein.

### Sampled Meta-Analytic Research Varies in Terms of Scientific Quality

The validity of information from empirical educational science rests on the appropriate application and reporting of research methodology to determine educational effectiveness. Consequently, systematic syntheses of research beyond summarizing results must also assess the scientific quality of the underlying research (Polanin et al., 2017). Based on 37 selected assessment criteria, our results demonstrate that the sampled meta-analyses overall meet the current quality criteria in meta-analysis research to a large extent. However, single meta-analyses also still vary in their adherence to the complete set of quality criteria. As high quality cannot be taken for granted, quality ratings of individual meta-analyses should be considered when interpreting aggregated effectiveness information. Low ratings imply that the recommended research methodology was not employed or sufficient reporting was not provided (or both). While the former can often lead to biased results (Borenstein et al., 2010), the latter at least impedes reproducibility and jeopardizes research progress (Polanin et al., 2020). However, despite some heterogeneity of the observed scientific quality in our sample, a substantial number of meta-analyses followed most of the guidelines (e.g., Fan et al., 2017; Schneider et al., 2018; van Alten et al., 2019). Along with guidance from standard documents (e.g., MARS) and recent publications (Pigott and Polanin, 2020), these can provide practical examples on how to adequately conduct and report meta-analytic research. Moreover, certain important criteria (e.g., search details, clear statement of inclusion criteria, analysis of publication bias) have been considered by a large majority of authors, thereby demonstrating additional improvement as compared to previous reviews (Ahn et al., 2012). Further, recent analyses of the quality of quantitative research synthesis in education and psychology (Schneider and Preckel, 2017; Polanin et al., 2020; Wedderhoff and Bosnjak, 2020) has revealed that our results are in line with the current practice in high-impact publication outlets. A few recurring issues in the literature as well as in our sample include insufficient reporting and accessibility of raw data—that is, coding information and insufficient application of meta-analytic methods to prevent biased results (Schneider and Preckel, 2017). Since none of the sampled publications was pre-registered and less than half (44%) of the publications provide sufficient data for replication, open research practices are still a matter of concern in research synthesis as they are in educational research more generally (Makel et al., 2021). This underlines the importance of efforts to facilitate preregistration of research synthesis for example by providing elaborated templates that specify information necessary for transparent reporting.

Importantly, the scientific quality of meta-analytic findings also rests on the quality of primary research. Even in their most advanced and differentiated form, the meta-analytic technique is limited by the number and quality of the primary studies to which it is applied (Lipsey and Wilson, 1993). This aspect deserves special attention as the so-called “garbage in–garbage out” problem has been around as long as meta-analytic research (see Eysenck, 1978); thus, the issue remains unresolved. A recent review (Wedderhoff and Bosnjak, 2020) on the assessment of primary study quality in quantitative reviews revealed that from among 225 meta-analyses published in *Psychological Bulletin* in the last 10 years, 40 (18%) considered quality differences in primary studies. Moreover, assessment strategies varied widely, which is attributed to a lack of a consensual operationalization of study quality. Considering that the underlying primary research of this review also demonstrates variation in terms of several quality indicators and that this variation can be associated with effect size variance (Cheung and Slavin, 2016; Lazonder and Harmsen, 2016), a more systematic investigation—that transcends testing single indicators as moderators and employs existing study quality assessment tools (e.g., Study DIAD, Valentine and Cooper, 2008)—is paramount for the further development of this evidence base.

Thus, echoing the concerns about the quality of research in education—both on a primary research and research synthesis level—(see Makel et al., 2021), our quality analysis demonstrates that despite increasing adherence to quality criteria in published meta-analyses, there is still considerable room for improvement. Initial action has been taken by the research community in providing standard documents, assessment tools, protocols, templates and websites (e.g., https://osf.io/) to increase transparency and quality. It is now up to researchers to make better use of these aids and guidelines in planning, conducting and reporting their research given their high responsibility for the research community but also for communities of practice who rely on their expertise and integrity.

### Limitations and Implications for Future Research

In this section, we describe salient limitations that warrant attention and further discussion in future research. Our conclusions extend a few of the general concerns and potential biases that are almost inherent in educational effectiveness research, such as general bias to overestimate the effects of new forms of instruction compared with regular forms (e.g., Ma et al., 2014; Schneider and Preckel, 2017).

One of the first limitations concerns the generalizability of our findings for current and international secondary mathematics and science education. Although most of the included meta-analyses were published within the last 5 years, there is still a considerable time-lag between the experiment that generated the primary data and the publication of this review. Within this time interval, the development of effective interventions and technologies has continued and studies that document this effectiveness have been published, which are not part of this review. This is a common concern in the review literature, which is more pronounced in systematic reviews of meta-analytic research (as a second-order synthesis), and in rapidly developing fields of research such as educational instructions (see Polanin et al., 2017). For future research, open and transparent study protocols and open data could facilitate the updating process for rapidly outdated meta-analyses (Pigott and Polanin, 2020; Polanin et al., 2020). Similar to research in higher education (Schneider and Preckel, 2017), a large proportion of included research stem from the United States, followed by a substantially fewer studies from other countries (see also Li et al., 2020). However, the results from several meta-analyses in our sample, demonstrate that the geographical origin of a study can significantly moderate effect sizes (e.g., Schroeder et al., 2017; Chen and Yang, 2019). This raises the question of how experimental research on educational effectiveness can be promoted in countries outside North America in order to enhance the generalizability of findings worldwide.

A second drawback concerns our reliance on results from statistical significance testing, particularly in moderator analyses. The authors in our sampled meta-analyses either used analogs to analysis-of-variance models to examine the moderating effects of single moderatos or meta-regression models to test multiple moderators and their association with effect-size variation in a single model. Both model types rely on statistical significance testing to ascertain whether or not a moderator effect is present. Since this systematic review utilizes information from moderator tests both for study selection and in reporting context-specific effectiveness information, we have implicitly accepted the criterion of statistical significance (at a 0.05 Alpha level) for crucial decisions in what we present as evidence. Although recently criticized, the practice of null-hypothesis statistical significance testing remains a dominant practice in the social sciences, and there is evidence from psychological research that statistical significance tests and Bayes factors as alternatives almost always agreed with regard to which hypothesis is better supported by the data (Wetzels et al., 2011). However, a common problem in meta-analyses—particularly in moderator tests—is the issue of low statistical power due to the small numbers of available effect sizes from primary research, which brings an increased likelihood of false negatives or type II errors when applying statistical significance tests (Cafri et al., 2010; Hempel et al., 2013). Consequently, our sample might suffer from the inappropriate inclusion of certain aggregated effect sizes, because the significance test failed to detect the presence of a moderator effect (by schooling level and/or subject domain), when the effect is actually present. Conversely, numerous meta-analyses in our sample conducted multiple univariate moderator tests without correcting Alpha levels, which raises concerns about the inflation of Type I error rates and increases the likelihood of falsely identified moderator effects (Polanin and Pigott, 2015). However, this practice is more a problem of the accurate application of statistical significance testing in moderator analysis rather than one of statistical significance testing *per se*.

A third limitation that warrants discussion concerns the usage of the presented findings as evidence in context-specific decision-making. Although meta-analytic findings are often praised for their usefulness to decision-makers—since they represent comprehensive summaries based on a robust database (e.g., Pigott and Polanin, 2020)—interventions adopted on the basis of this aggregated evidence often fail to be effective in practice. According to Joyce and Cartwright (2020), this is not surprising, as findings based on one or several experimental studies provide evidence that the intervention worked in the past (causal ascription) but no evidence that the intervention will work in a specific context in the future (local effectiveness prediction). Nevertheless, findings related to aggregated positive effectiveness for a certain context play a role in supporting a prediction, as they indicate that the intervention *can* produce the effect under more or less similar sets of circumstances. A targeted collection of such indications is where we believe the contribution of this review lies. Even though we selected and summarized this information not only for the research community, but also to address practitioners as e.g., teachers and teacher educators, it seems clear that these non-specialist audiences often face challenges in accessing and interpreting current research (see Diery et al., 2020, 2021). One way to offer support is to provide some supportive services which select and translate research for non-specialist audiences. Whereas the selection part is mainly described by this contribution, for the translation part we have established an online service platform that provides plain language summaries for meta-analyses which are selected and included in this review (Seidel et al., 2017a,b). This service, funded by the German ministry of education and research, can be accessed by any teacher and teacher educator free of charge via http://www.clearinghouse-unterricht.de. The website additionally includes a glossary and other educative material to empower practitioners in order to help them adequately interpret research evidence.

## Conclusion

Through this systematic review of meta-analyses, we put forward a multiple steps approach to determine an evidence base for a particular field of educational practice. As a first step we chose effective teaching as a prominent field of educational practice. Since targets in teaching are provided on the level of a certain subject *and* educational level, we argued that effectiveness information that cuts across these two categories for specification is best suitable for informing the practice of effective teaching. In this regard, our study is the first to provide and apply a heuristic for filtering the best available effectiveness information based on such a context specification. Our results from the field of secondary mathematics and science teaching demonstrate that context-specific effect sizes information may often differ from more general effect size information on teaching effectiveness. Although our findings indicate that there is substantial amount of relevant and encouraging context-specific information available they also show that we had to exclude many studies because they did not offer information generalizable to this specific context. Thus, although meta-analytic research has strongly developed over the last few years, providing context-specific and high-quality evidence still needs to be a focus in the field of secondary mathematics and science teaching and beyond. This systematic review could offer guidance and encouragement on this continuous path.

## Data Availability Statement

Publicly available datasets were analyzed in this study. This data can be found online at: https://osf.io/9n99n/?view_only=bb30c83e9bf34d73a79138ddcf91da5c and in the supplements of this article.

## Author Contributions

MK and AH developed the coding scheme and carried out literature search and coding. MK wrote the first draft of the manuscript. All authors contributed to the conception of the review, the manuscript revision, read, and approved the submitted version.

## Funding

The present study was conducted as part of the Project Clearinghouse on Effective Teaching (www.clearinghouse-unterricht.de), funded by the German Federal Ministry of Education and Research (01JA1801).

## Conflict of Interest

The authors declare that the research was conducted in the absence of any commercial or financial relationships that could be construed as a potential conflict of interest.

## Publisher's Note

All claims expressed in this article are solely those of the authors and do not necessarily represent those of their affiliated organizations, or those of the publisher, the editors and the reviewers. Any product that may be evaluated in this article, or claim that may be made by its manufacturer, is not guaranteed or endorsed by the publisher.

## Footnotes

^1^We employ the term “strategy” to delineate all kinds of instructional interventions, ranging from multicomponent programmes to specific instructional approaches and practices that can be adopted by teachers to support student learning.

^2^This specification allows for multiple types of study designs related to the experimental research paradigm.

^3^For details on the operationalization of specificity, please consult the following section on “extraction of effect sizes”.

^4^In their meta-analysis on homework and student achievement, Fan et al. (2017) used an aggregated correlation coefficient r to provide estimates on how different homework practices relate to student achievement outcomes.

^5^Statistical average for individual meta-analyses.

^6^See Table 1 for category description.

^7^Although several meta-analyses contain some of the same studies, we decided to retain all selected meta-analyses and effect sizes in our summary for several reasons. First, the overlap of primary studies is limited to only a few meta-analyses. Second, the overlap is usually small and concerns only a few primary studies. Third, although some meta-analyses include some of the same studies, they adopt a different focus of analysis. Fourth, our aim is to provide an overview of the existing meta-analyses and effect sizes for secondary mathematics and science teaching and not to conduct a second-order meta-analysis.

^8^Our sampled meta-analyses did not report any negative context-specific effect size (see Table 2). For 13 out of 78 (17%) context-specific effect sizes, statistical significance levels exceeded conventional thresholds (*p* > 0.05).
