# Supplementary material for: Determining an Evidence Base for Particular Fields of Educational Practice: A Systematic Review of Meta-Analyses on Effective Mathematics and Science Teaching
Source: Front Psychol. 2022 Apr 25;13:873995. doi: 10.3389/fpsyg.2022.873995 (PMC9083191; doi:10.3389/fpsyg.2022.873995)
Supplement: Supplementary file 1 [file Data_Sheet_1.PDF]

S1 Search Details

Search Strategy

I. Search Criteria

A. Inclusion Criteria

1. Meta-Analysis
2. Includes Mathematics and Science education
3. Includes Secondary Education population
4. Effects on student outcomes
5. Published since 2004
6. Published in English
7. Peer reviewed

B. Exclusion Criteria

1. Focus on Special Students (e.g.students with disabilities, gifted students, math difficulties, at-risk students)
2. Context-specific phenomena (e.g. charter schools USA, single countries, country comparisons, state comparisons)
3. Influence of non-instructional factors (parental involvement, music, sports / physical activities)
4. Out of school learning

II. Step 1: Database Search

A. Selection of Databases

- Web of Science (TS or TI)
- Scopus (TITLE-ABS-KEY)
- ERIC, PsycInfo, PsyIndex (EbscoHost) (AB or TI)

B. Search term combination

(classroom OR school* OR student* OR teach* OR learn* OR education* OR instruc* OR inquiry)

AND

(STEM OR Engineer* OR Chemistry OR Biolog* OR Geograph* OR Math* OR Science* OR Physic* OR Technolog*)

AND

(skill* OR competen* OR abilit* OR achiev* OR attain* OR aptitude* OR perform* OR learn* OR know* OR understand* OR expertise OR comprehen* OR assess* OR concept* OR proficien* OR development* OR success OR persist*)

AND (meta-analy*)

NOT (special education OR disabil* OR special need*)

C. Search strings

Database	Search String
Web of science (SSCI)	((TS=((classroom OR school* OR student* OR teach* OR learn* OR education* OR instruc* OR inquiry)) AND TS=((STEM OR Engineer* OR Chemistry OR Biolog* OR Geograph* OR Math* OR Science* OR Physic* OR Technolog*)) AND TS=((skill* OR competen* OR abilit* OR achiev* OR attain* OR aptitude* OR perform* OR learn* OR know* OR understand* OR

	expertise OR comprehen* OR assess* OR concept* OR proficien* OR development* OR success OR persist*)) AND TS=((meta-analy*)) NOT TS=((special education OR disabil* OR special need*))) OR ((TI=((classroom OR school* OR student* OR teach* OR learn* OR education* OR instruc* OR inquiry)) AND TI=((STEM OR Engineer* OR Chemistry OR Biolog* OR Geograph* OR Math* OR Science* OR Physic* OR Technolog*)) AND TI=((skill* OR competen* OR abilit* OR achiev* OR attain* OR aptitude* OR perform* OR learn* OR know* OR understand* OR expertise OR comprehen* OR assess* OR concept* OR proficien* OR development* OR success OR persist*)) AND TI=((meta-analy*)) NOT TI=((special education OR disabil* OR special need*))))) AND LANGUAGE: (English) AND DOCUMENT TYPES: (Article)
Scopus	TITLE-ABS-KEY (classroom OR school* OR student* OR teach* OR learn* OR education* OR instruc* OR inquiry) AND TITLE-ABS-KEY (stem OR engineer* OR chemistry OR biolog* OR geograph* OR math* OR science* OR physic* OR technolog*) AND TITLE-ABS-KEY (skill* OR competen* OR abilit* OR achiev* OR attain* OR aptitude* OR perform* OR learn* OR know* OR understand* OR expertise OR comprehen* OR assess* OR concept* OR proficien* OR development* OR success OR persist*) AND TITLE-ABS-KEY (meta-analy*) AND NOT TITLE-ABS-KEY (special education OR disabil* OR special need*) AND PUBYEAR > 2000 AND (LIMIT-TO(SUBJAREA,"SOCI") OR LIMIT-TO(SUBJAREA,"PSYC")) AND (LIMIT-TO(LANGUAGE,"English")) AND (LIMIT-TO(SRCTYPE,"j"))
ERIC, Psycinfo, Psyndex (EbscoHost)	TI ((classroom OR school* OR student* OR teach* OR learn* OR education* OR instruc* OR inquiry) AND (STEM OR Engineer* OR Chemistry OR Biolog* OR Geograph* OR Math* OR Science* OR Physic* OR Technolog*)

AND (skill* OR competen* OR abilit* OR achiev* OR attain* OR aptitude* OR perform* OR learn* OR know* OR understand* OR expertise OR comprehen* OR assess* OR concept* OR proficien* OR development* OR success OR persist*) AND (meta-analy*) NOT (special education OR disabil* OR special need*)) OR AB ((classroom OR school* OR student* OR teach* OR learn* OR education* OR instruc* OR inquiry) AND (STEM OR Engineer* OR Chemistry OR Biolog* OR Geograph* OR Math* OR Science* OR Physic* OR Technolog*) AND (skill* OR competen* OR abilit* OR achiev* OR attain* OR aptitude* OR perform* OR learn* OR know* OR understand* OR expertise OR comprehen* OR assess* OR concept* OR proficien* OR development* OR success OR persist*) AND (meta-analy*) NOT (special education OR disabil* OR special need*)) OR KW ((classroom OR school* OR student* OR teach* OR learn* OR education* OR instruc* OR inquiry) AND (STEM OR Engineer* OR Chemistry OR Biolog* OR Geograph* OR Math* OR Science* OR Physic* OR Technolog*) AND (skill* OR competen* OR abilit* OR achiev* OR attain* OR aptitude* OR perform* OR learn* OR know* OR understand* OR expertise OR comprehen* OR assess* OR concept* OR proficien* OR development* OR success OR persist*) AND (meta-analy*) NOT (special education OR disabil* OR special need*))

III. Step 2: Hand Search

Top Review Journals in Education

- Review of Educational Research
- Educational Research Review
- Review of Educational Psychology

Top Journals in Education

- Journal of Educational Psychology
- Learning and Instruction
- Computers & Education
- Journal of Teacher Education
- American Educational Research Journal
- Journal of the Learning Sciences

Top Journals in Science Education

- Journal of Research in Science Teaching
- Science Education
- Journal of Science Education
- International Journal of Science Education

IV. Ancestral approach

- Check the included MAs for relevant cited work
- Check for duplicates in the database

V. Integration & Removal of Duplicates

- Download the results of the data bases

- Delete duplicates
