# Supplementary material for: Determining an Evidence Base for Particular Fields of Educational Practice: A Systematic Review of Meta-Analyses on Effective Mathematics and Science Teaching
Source: Front Psychol. 2022 Apr 25;13:873995. doi: 10.3389/fpsyg.2022.873995 (PMC9083191; doi:10.3389/fpsyg.2022.873995)
Supplement: Supplementary file 2 [file Data_Sheet_2.PDF]

Table S2a

Characteristics of included meta-analyses (N = 41) – Publication outlets

Journal	n of meta-analyses
Educational Research Review	9
Review of Educational Research	7
Educational Psychology Review	5
Journal of Educational Psychology	4
Computers & Education	2
Journal of Research in Science Teaching	2
Journal of Computer Assisted Learning	2
Other (1x) [a]	10

Note. n = number

[a] Journals appearing once: School Science and Mathematics; Metacognition and Learning; NASSP Bulletin; Developmental Review; IAFOR Journal of Education; Asia Pacific Education Review; International Journal of Educational Research; Educational Technology Research and Development; International Education Studies

Table S2b

Characteristics of included meta-analyses (N = 41) – Year of Publication

Year of Publication	Number of meta-analyses
2005	1
2006	0
2007	1
2008	1
2009	0
2010	2
2011	0
2012	2
2013	5
2014	3
2015	3
2016	6
2017	8
2018	6
2019	3

Table S2c

Characteristics of included meta-analyses (N = 41) – Time span covered

Time span between first and last primary study included (in years)	Number of meta-analyses
< 10	5
10-19	17
20-29	12
30-39	2
> 39	5

Table S2d

Characteristics of included meta-analyses (N = 41) – Publication delay

Delay between last study included and publication of meta-analysis (years)	Number of meta-analyses
1	8
2	13
3	12
4	3
5	4
> 5	1
